# The effectiveness of family counselling on reducing exposure to secondhand smoke at home among pregnant women in Iran

**DOI:** 10.18332/tpc/113105

**Published:** 2019-11-15

**Authors:** Farzaneh Soltani, Fariba Barzegar, Gita Sangestani, Ghodratolah Roshanaii, Azam Maleki

**Affiliations:** 1 Mother and Child Care Research Center, Hamadan University of Medical Sciences, Hamadan, Iran; 2 Student Research Center, Hamadan University of Medical Sciences, Hamadan, Iran; 3 Modeling of Non-CommunicableDiseases Research Center, Hamadan University of Medical Sciences, Hamadan, Iran; 4 Social Determinants of Health Research Center, Zanjan University of Medical Sciences, Zanjan, Iran

**Keywords:** health behavior, smoking, pregnant women, secondhand smoke, smoking reduction

## Abstract

**INTRODUCTION:**

Pregnant women are often exposed to secondhand smoke that affects them and their child. Our aim was to determine the effectiveness of family counselling using the BASNEF model on reducing exposure to secondhand smoke at home among pregnant women.

**METHODS:**

A quasi-experimental study was conducted on 103 pregnant women exposed to secondhand smoke. They were selected using a multi-stage cluster sampling method and allocated into intervention (50 people) and control (53 people) groups. Four family counseling sessions using the BASNEF model were held for the intervention group while the control group received routine care. The outcomes were measured before and at one month after the last session of counselling.

**RESULTS:**

In the timeframe before the intervention, the number of days in which there was reported exposure to secondhand smoke was 5.08 ± 1.1 in the intervention group, significantly decreasing to 3.5 ± 1.6 after the intervention (p<0.001). No significant change was observed in the control group (p=0.1). Also, the mean scores of all constructs of the BASNEF model increased significantly after the intervention compared to those of the control group (p<0.05).

**CONCLUSIONS:**

Family counseling had a positive effect on decreasing the exposure to secondhand smoke at home among a sample of pregnant women. The BASNEF model is useful for implementing educational care programs in these settings.

## INTRODUCTION

Exposure to secondhand smoke (SHS) can be through many sources and is linked to adverse health effects^1,2^. Among those exposed to SHS, women and especially pregnant women have been studied widely because of the impact SHS exposure may have on the growing fetus^3,4^. A study among pregnant women in Greece showed that 72% of women are exposed to SHS in the home and 64% in the workplace^5^. Also, SHS is associated with effects such as spontaneous abortion, preterm birth, and small for gestational age^6,7^. Research has shown that the weight, height and head circumference of newborn babies born from mothers exposed to smoke during pregnancy are lower than those of infants whose mothers were not exposed to SHS^8^. Research further suggests that cognitive and behavioural control disorders in elementary students may be attributable to exposure to smoking before and after childbirth^9,10^.

The extent of exposure to SHS during pregnancy depends on various socioeconomic and cultural conditions^5^. In Iran, women smoke less than men, but the high cigarette smoking rates in men, especially in the western regions of the country^11,12^, along with a close family life with relatives, have increased the risk of exposure to SHS for pregnant women. Designing effective interventions for creating a relatively healthy environment for pregnant women and protecting them from adverse maternal and fetal outcomes are among the priorities of healthcare services for pregnant women. Fortunately, pregnancy in all cultures provides an opportunity to change the behaviour of the family, especially when it comes to the health of the infant^12,13^.

Since a change in the behaviour of the smoker spouse is the most effective and logical intervention to reduce the exposure of pregnant women to SHS, the present study was carried out using comprehensive and well-known beliefs, attitudes, subjective norms and enabling factors model (BASNEF), based on which individual’s beliefs about the outcomes and benefits of a healthy behaviour are very useful in creating their attitudes towards that behavior. Opinions of other people who live with them are essential and play a significant role in creating the subjective norms towards the desired behaviour^14-16^. BASNEF is a comprehensive and complete model that is adopted to study behaviours, offers plans for change, and defines the factors effective for the individual’s decision making^14^.

The aim of the study was to determine the effectiveness of family counselling based on BASNEF model on the times pregnant women are exposed at home to secondhand smoke.

## METHODS

### Sampling, setting and participants

**Figure 1:**
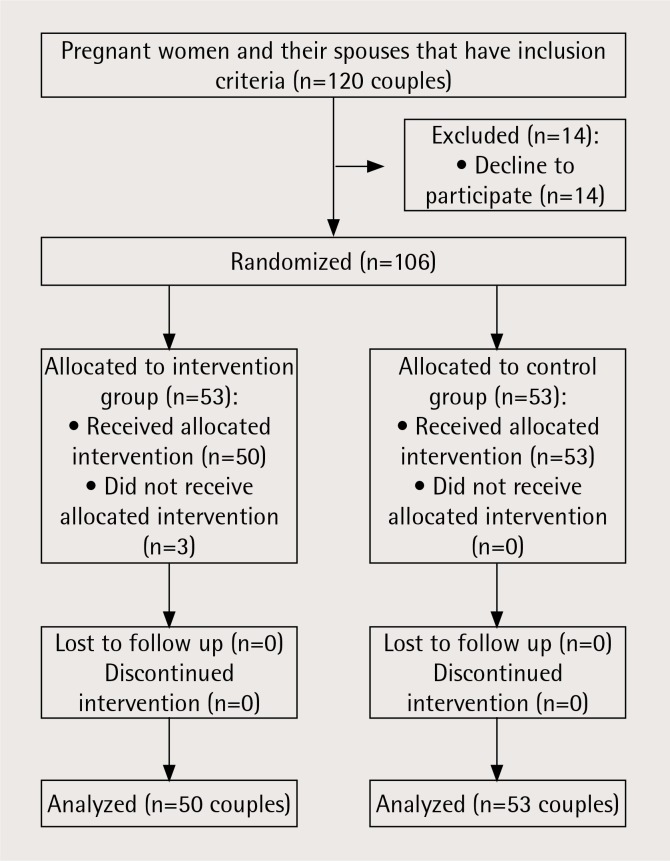
Flow chart of the study

This quasi-experimental study was conducted on 103 pregnant women referred to health centres in Hamadan, Iran, and their smoker spouses. The research population consisted of pregnant women exposed to SHS who attended health centres for prenatal care in Hamadan from October 2017 to May 2018. The samples were drawn through a multi-stage cluster sampling method in two steps. In the first step, Hamadan city was divided into three geographical regions and from each 2 health centres were chosen randomly from a list of health centres; thus 6 health centres were included in the present study. In the second step, one health centre in each region was allocated to the intervention group and the other was allocated to the control group, randomly, to prevent information transfer between the two groups. Participants were chosen randomly among the pregnant women who were eligible for inclusion using the Prenatal Care Information System so that in each health centre 20 people (totally 120 couples) were selected for participation in the study (Figure 1).

The inclusion criteria were the presence of a smoker spouse, healthy singleton pregnancy, and gestational age less than 30 weeks. The exclusion criteria were the presence of medical and obstetric complications, intra-uterine fetal death, divorce, change of residence, and the absence of more than one counselling session.

### Instruments

Data were collected using a demographic questionnaire, and self-constructed questionnaire designed using a similar study for assessing BASNEF model constructs^16^. Self-constructed questionnaire was designed in 5 subscales: 1) spouse’s knowledge about the harms of exposure to cigarette smoke in pregnant women and their child, 9 items with three Likert spectrums; 2) spouse’s attitude towards reducing smoking in the presence of his pregnant wife, 13 items with five Likert spectrums; 3) subjective norms about reducing smoking in the presence of pregnant women, 7 items with three Likert spectrums; 4) enabling factors of reducing smoking in the presence of pregnant women, 10 items with two Likert spectrums; and 5) behavior outcome evaluation, 6 items with two Likert spectrums. The times of smoking exposure at home were determined using an open-ended questionnaire. The validity of the questionnaires was confirmed through content validity method by eight academic members in reproductive health. Also, using Cronbach’s alpha for knowledge items (α=0.72), attitude items (α=0.70), subjective norms items (α=0.9), enabling factors items (α=0.83), behavioral evaluation (α=0.79) and behavioral intention (α=0.79), the reliability of the questionnaire was approved.

### Ethical considerations

This study was approved by the Ethics Committee of Hamadan University of Medical Sciences (IR. UMSHA.REC.1394.254). Participants were informed about the objective of the study, written consent was obtained from each participant, and they were assured of the confidentiality of information. In addition, participants could be excluded whenever they did not want to participate in the study.

### Intervention

The selected pregnant women and their spouses were invited through phone calls for a briefing session wherein written consent to participate in the study was obtained. The questionnaires were completed by pregnant women and their spouses. After necessary coordination, four weekly counselling sessions were held for the intervention group in groups of up to 10 people for 45–60 minutes, including 45 min of counselling and 15 min of questions and answers. The consultation sessions in the training class at the health centres were conducted by a researcher (PhD in Reproductive Health and Master of Science in Midwifery). The content of the intervention was family counselling in the form of lectures, group discussion, brainstorming, and questions and answers. Also, educational brochures related to the harms and adverse effects of smoking on pregnant women and unborn children were distributed. The control group received routine prenatal care and the time for completion of questionnaires was set. One month after the end of the intervention, the questionnaires were completed again by the two groups.

### Statistical analysis

Data were analyzed using SPSS software version 16. The Kolmogorov-Smirnov test was used to ensure the normal distribution of data. Chi-squared test, independent t-test and paired t-test were used to investigate the hypotheses. The significance level was considered <0.05.

## RESULTS

The mean age of the mothers in the intervention group was 28.2 ± 5.7 years and for the control group it was 29.5 ± 6.4 years; the corresponding ages for the spouses were 36.9 ± 11.0 years for the intervention group and 40.1 ± 12.1 years for the control group. There was no significant difference between the age of pregnant women and their spouses in both control and intervention groups. As noted in Table 1, pregnant woman’s employment and education, housing status, the number of smoking individuals at home, spouse’s education, duration (years) of spouse’s cigarette smoking, spouse’s employment, and the number of cigarettes smoked per day by the spouse, did not statistically differ between the two groups.

**Table 1:** Comparison of demographic characteristics between intervention and control groups, Iran, 2018 (n=103)

*Variables*		*Intervention*		*Control*		*p*
		*Frequency*	*%*	*Frequency*	*%*	
**Pregnant woman’s employment**	Employed	1	2	7	13.2	0.09
	Housewife	49	98	46	86.8	
**Pregnant woman’s education**	Illiterate	0	0	4	7.5	0.11
	Middle school	30	60	10	18.9	
	Diploma	16	32	22	41.5	
	University	4	8	17	32.07	
**Housing status**	Owner	40	80	42	79.2	0.92
	Rental	10	20	11	20.8	
**The number of smoking individuals at home**	Spouse	44	88	39	73.6	0.06
	Father-in-law	6	12	9	17	
	Brother-in-law	0	0	5	9.4	
**Spouse’s education**	Illiterate	4	8	7	23.2	0.55
	Middle school	27	54	25	47.2	
	Diploma	14	28	12	22.6	
	University	5	10	9	16.9	
**Duration of spouse’s cigarette smoking in years**	1–10	31	62	30	56.6	0.36
	11–21	13	26	10	18.9	
	22–32	6	12	10	19.9	
	≥32	0	0	3	5.6	
**Spouse’s employment**	Self-employed	46	92	45	84.9	0.26
	Public servant	4	4	8	15.1	
**The number of cigarettes smoked by the spouse per day**	1–10	19	38	19	37.3	0.76
	11–20	26	52	29	56.9	
	22–32	2	4	2	3.9	
	32–40	3	6	1	2	

Table 2 shows that after the intervention, the mean number of exposure times to SHS between the intervention and follow-up at home decreased significantly in the intervention group compared to the control group (p<0.001).

**Table 2:** Comparison of number of exposure times of pregnant woman to SHS at home inter and intra two groups before and after intervention, Iran, 2018 (n=103)

*Group*	*Before intervention*	*After intervention*	*Statistic (paired t-test)*	*p*
	*Mean ± SD*	*Mean ± SD*		
**Intervention**	5.08 ± 1.1	3.5 ± 1.66	9.3	<0.001
**Control**	4.02 ± 1.5	3.7 ± 1.56	1.4	0.1
**Statistic (independent t-test)**	0.63	4.1		
**p**	0.4	<0.001		

Table 3 shows that after the intervention, there were significant differences between the mean scores of behavioral intention of the spouse to reduce smoking at home, knowledge, attitude, enabling factors, and subjective norms in the intervention group compared to the control group, all statistically significant (p=0.04). After the intervention there were significant differences between the mean scores of all constructs of the BASNEF model in the intervention group (p<0.05) but these differences were not significant in the control group (p>0.05).

**Table 3:** Comparison of scores of BASNEF model constructs inter and intra two groups before and after intervention, Iran, 2018 (n=103)

*Variables*		*Before intervention*	*After intervention*	*Statistic (paired t-test)*	*p*
		*Mean ± SD*	*Mean ± SD*		
**Knowledge**	Intervention	3.70 ± 2.40	6.60 ± 1.94	6.07	<0.001
	Control	4.3 ± 1.551	4.94 ± 2.01	0.53	0.29
	Statistic (independent t-test)	1.52	4.24		
	p	0.07	<0.001		
**Attitude**	Intervention	28.16 ± 7.97	32.58 ±5.42	1.8	0.04
	Control	23.83 ± 6.78	23.92 ± 9.02	1.83	0.06
	Statistic (independent t-test)	0.92	0.18		
	p	0.18	<0.001		
**Enabling factors**	Intervention	7.86 ± 1.959	8.96 ± 1.653	3.65	0.001
	Control	7.45 ± 1.853	7.49 ± 1.815	0.54	0.11
	Statistic (independent t-test)	1.1	4.28		
	p	0.14	<0.001		
**Subjective norms**	Intervention	17.2 ± 2.143	18.82 ± 1.511	2.23	0.03
	Control	17.36 ± 2.739	17.19 ± 2.370	0.37	0.07
	Statistic (independent t-test)	0.7	4.13		
	p	0.5	<0.001		
**Behavioral intention of spouse to reduce smoking**	Intervention	3.44 ± 1.829	5.76 ± 1.791	6.5	<0.001
	Control	3.08 ± 2.005	3.00 ± 1.850	0.21	0.58
	Statistic (independent t-test)	0.95	7.68		
	p	0.17	<0.001		

## DISCUSSION

Our results indicate that family counselling using the BASNEF model has a positive impact on decreasing the times the intervention group where exposed to SHS at home compared to the control group. Enabling factors such as the provision of adequate information, the attitude of the pregnant woman’s spouse towards the harmful effects of cigarette smoking on pregnancy outcomes, and identification of subjective norms such as advising other people about reducing smoking at home improved behavioural intention of spouses.

Other consistent studies have supported that behavioural change interventions can lead to increased awareness of harms of exposure to smoking and increased sensitivity of people to reduce exposure to SHS at home^17,18^. Studies in this area are faced with many limitations such as adapting these models to different countries and conditions^19^. Karimiankakolaki et al.^4^ in their study have shown that an educational intervention aiming at reducing SHS exposure of pregnant women could be improved by the level of knowledge, attitude, self-efficacy, and practices of men. The present study showed that if men are involved in safe parenting programs, they can protect the family’s health and participate actively in the role of a responsible father to ensure the woman’s health during pregnancy. Fathers need to make some changes to adapt to this new role, which is sometimes more difficult because their potential role is often underestimated by their relatives and even healthcare providers^20^. It should be noted that the participation of spouses in ensuring the health of pregnant women is a process of social and behavioural change needed to be created in men so that they undertake more responsibility for the health of mothers and children.

The participation of men in providing a healthy environment for their pregnant women, especially in developing countries where men are often decision-makers at the family and community level, is important^21^. The need for training to increase participation of fathers in prenatal care resulting in positive effects on maternal and infant health has been shown in numerous studies in different countries^22^. However, it should be noted that the training of men does not guarantee support and participation of spouses during the pregnancy period; changing their viewpoint, attitude and behaviour requires more effective interventions^23^. Thus, the use of behavioural theories in educational interventions can improve the effectiveness of these interventions. In fact, one can be trained to change unhealthy behaviour, but choosing a mentality norm is done by the individual himself and influenced by family, friends, relatives and society. This means that the person evaluates the new behaviour with the level of approval or rejection of those who are important to him, and considering attitudes towards the behaviour and subjective norms leads to decision making in order to adopt the new behaviour^24^. In the meantime, the direct contribution between enabling factors and, in particular, healthcare providers with awareness and behavioural intention, clearly confirms the vital role of these factors in reinforcing preventive behaviours^25^.

### Limitations

This study has some limitations. One of the limitations is the short follow-up interval that was limited to one month. Further studies are recommended to be conducted with longer follow-up intervals. The relatively small sample size was another limitation as it substantially reduced statistical power. Finally, it was quasi-experimental research, so its internal validity may have declined due to the lack of random assignment.

## CONCLUSIONS

Family counselling had a positive effect in decreasing the times of secondhand smoke exposure at home in pregnant women. In addition, using the BASNEF model can be acceptable for implementing educational care programs. Participation of men in maternal and child care provides new opportunities for healthcare providers to train future fathers to ensure a healthy environment for their family.
